# Combination effects of nitrite from fermented spinach and sodium nitrite on quality characteristics of cured pork loin

**DOI:** 10.5713/ajas.18.0903

**Published:** 2019-02-07

**Authors:** Tae-Kyung Kim, Mi-Ai Lee, Jung-Min Sung, Ki-Hong Jeon, Young-Boong Kim, Yun-Sang Choi

**Affiliations:** 1 Research Group of Food Processing, Korean Food Research Institute, Wanju 55365, Korea; 2 World Institute of Kimchi an Annex of Korea Food Research Institute, Gwangju 61755, Korea

**Keywords:** Spinach, Natural Nitrite, Synthetic Nitrite, Cured Pork Loin

## Abstract

**Objective:**

The purpose of this study was to investigate the effect of fermented spinach derived nitrite and sodium nitrite on cured pork loin.

**Methods:**

The following treatments were prepared using brine (8% [w/v] salt): Control (−), no nitrite added; Control (+), 0.08% (w/v) sodium nitrite brine; T1, 0.04% (w/v) nitrite fermented spinach juice in 0.04% (w/v) sodium nitrite brine; T2, spinach juice in 0.04% (w/v) sodium nitrite brine; T3, 0.04% (w/v) nitrite fermented spinach juice used as sodium nitrite free brine; and T4, spinach juice used as sodium nitrite free brine. T2 and T4 were incubated to allow to reduce nitrate to nitrite.

**Results:**

Spinach juice did not affect cooking loss and pH but negatively influenced flavor and overall acceptability (p<0.05). T1 samples containing synthetic and natural nitrites showed the highest redness values. Spinach juice negatively affected volatile basic nitrogen; however, thiobarbituric acid reactive substance values of T1 and T3 were similar to those of controls (+) (p>0.05). Residual nitrite content decreased with decreasing synthetic nitrite levels. T1 and control samples showed no significant differences in overall acceptability (p>0.05).

**Conclusion:**

Thus, combined synthetic and natural nitrites improved the quality of cured pork loin.

## INTRODUCTION

Consumer demands for healthy and natural-friendly meat products is on the rise [1]. To satisfy the increasing consumer demands for natural meat products, the meat industry has increasingly utilized natural additives that was considered as healthy foods to consumer [2]. As a result, several established chemical additives such as sodium nitrite, sodium polyphosphate, and butylated hydroxytoluene have been replaced with natural additives to enhance the quality characteristics of meat and dairy products [3,4]. Natural antioxidants, colorants, emulsifiers, and spices, such as green tea, isolated soy bean powder, and cochineal, have been used as substitutes for chemical additives [5,6].

In general, nitrite has been used as an antioxidant, flavor enhancer, and color enhancer in meat [7] and synthetic nitrite was produced with various methods such as scrap iron filing, reaction of nitrogen oxides in alkaline solution [8]. In particular, natural nitrite derived from celery, radish, cabbage, and soil, and water was used to replace sodium nitrite [9]. Nitrates from some vegetables such as celery can pass through the nitrogen cycle in microorganisms, which reduce nitrates to nitrites and this nitrite source can used in the processing of meat and meat products [9].

Krause et al [10] reported that curing procedure with brine containing vegetable juice can serve as a substitute for the conventional curing procedure. Furthermore, the properties of meat cured with vegetable juice and starter culture were comparable to those of meat cured with sodium nitrite [11]. Celery powder has been used commercially as a natural curing agent because it does not produce an off-flavor in the cured meat [9,12]. In addition, meat cured with celery showed no significant differences in quality characteristics with traditionally cured meat products. However, celery can trigger allergic reactions even after heating at 100°C [13].

According to Sebranek and Bacus [12], spinach juice contains 3,227 ppm nitrate. Spinach has a high nitrate content and can therefore be used as a natural source of nitrite. Furthermore, spinach contains high levels of antioxidants, which can ameliorate the effects of a high-fat and high-cholesterol diet by reducing the risk of oxidative stress related disease [14]. Therefore, spinach can serve as a good natural nitrite source to improve the quality of meat and meat products.

The purpose of this study was to investigate synergistic effects of sodium nitrite and natural nitrite on the quality characteristics of cured pork loin and to develop a method of utilizing natural nitrite from spinach for the curing of meat.

## MATERIALS AND METHODS

### Preparation of the curing agent and processing of cured pork loins

Spinach powder was purchased from a local market. Spinach juice was prepared by mixing 100 g of spinach with 1,000 mL of distilled water. Spinach juice was fermented by the nitrate reducing starter culture (S-B-61, Bactoferm TM, Chr. Hansen Inc., Gainesiville, FL, USA) and subsequently incubated at 30°C for 24 h in a shaker incubator and then filtered by Whatman No 1. The fermented spinach juice was then used as a nitrite substitute. Fermented spinach juice (pH, 5.32; L*, 25.46; a*, 0.78; b*, 4.91; nitrite content, 20,232 ppm) was stored in amber flasks in the dark at 4°C and was used within 24 h. The 10% (w/v) of filtered spinach juice (pH, 5.76; Commission Internationale de l’Eclairage [CIE] L*, 31.72; CIE a*, 12.28; CIE b*, 24.18) was prepared as a nitrate substitute for the curing step [15] and all brine of treatments was stored at 4°C in the dark refrigerator until used within 24 h.

Fresh pork loins (*Musculus longissimus thoracis*) were purchased from a local processor and excess fat and connective tissues were trimmed. The center portion of pork loins was cut into 6 to equal thickness of one-inch slices and 42 slices were subjected to all treatments after mixed randomly (7 loins were used in one replicate to all treatments). Prepared sliced pork loins were pickled randomly in brine (8% salt, nitrite concentrate was 0.08%) and the volume of brine which was used as pickling solution was 40% of meat weight. After measured the nitrite concentration of fermented spinach juice, fermented spinach juice were diluted to a final nitrite concentration of 0.08% of brine with distilled water and spinach juice that was not fermented was added in brine as same level between fermented spinach juice. The formulations of the brine solutions are presented in Table 1. Samples from the different treatments were cured for 4 days in a refrigerated room at 4°C [16]. T2 and T4 that cured for 84 h in spinach juice brine were incubated for 12 h at 15°C in low temperature circulating water bath (SLTC22, LK lab, Namyangju, Korea) with starter culture to reduce nitrate to nitrite. After curing and incubation, the cured pork loins were heated for 30 min at 75°C in a chamber (MAXi3501, Kerres, Backnang, Germany) and subsequently cooled at room temperature (25°C) for 1 h. All experiments was done in a day.

### Cooking loss

The changed weight before and after heating procedure that was proceeded for 30 min at 75°C was measured and calculated to cooking loss (%). After heating, samples were cooled at room temperature (25°C) for 1 h [15].

### pH measurement

Raw and cooked cured pork loins (5 g) were added with 20 mL of distilled water and homogenized. The pH values of the raw and cooked samples were then measured using a pH meter (Model 340, Mettler-Toledo GmbH, Greifensee, Switzerland) calibrated with pH 4.0, 7.0, 10.0 solution at 25°C. Raw samples were stored at 4°C prior to pH analysis and cooked samples were measured after cooling for 1 h at 25°C.

### Color evaluation

The internal surface characteristics of the raw or cooked cured pork loin samples were determined using a 10 mm colorimeter (Minolta Chroma meter CR-210, Minolta Ltd., Tokyo, Japan, calibrated with a white plate, L* value: 97.83, a* value: 0.43, b* value: 1.98, 2° observer, D_65_) after blooming for 30 min at 4°C. Color of spinach juice and fermented spinach juice placed on the 1 cm plastic dish used as light port was measured using a colorimeter calibrated with a white plate under plastic dish. Hue angle (H°), color difference (ΔE*), and chroma difference (ΔC*) were calculated using the following equations: H° = arctan (b*/a*), ΔE* = (ΔL*^2^+Δa*^2^+ Δb*^2^)^1/2^, and ΔC* = (Δa*^2^+Δb*^2^)^1/2^ [15].

### Thiobarbituric acid reactive substances evaluation

Lipid oxidation was assessed following the distilled 2-thiobarbituric acid (TBA) method with minor modifications [17]. Each 10-g cooked sample was added with 50 mL of distilled water, homogenized for 2 min, and transferred to a distillation tube. The cup used for blending was washed with an additional 47.5 mL of distilled water, and the solution was subsequently added to the same distillation flask containing 2.5 mL of 4 N HCl and a few drops of an antifoam agent, silicone o/w (KMK-73, Shin-Etsu Silicone Co., Ltd., Tokyo, Japan). The resulting mixture was distilled, and 50 mL of the distillate was collected. Five mL of 0.02 M TBA in 90% acetic acid (TBA reagent) was added to a vial containing 5 mL of the distillate, and the resulting mixture was and mixed thoroughly. The vials were capped and heated in a boiling water bath (100°C) for 30 min to develop the chromogen, and the mixture was cooled to room temperature (25°C). The absorbance was measured at 538 nm against a blank solution containing 5 mL of distilled water and 5 mL of TBA-reagent using a UV/VIS spectrophotometer (Optizen 2120 UV plus, Mecasys Co. Ltd., Daejoen, Korea). Thiobarbituric acid-reactive substances (TBARS) content was calculated based on a standard curve (8 to 50 nmol) of malondialdehyde, which was freshly prepared by acidification of 1,1,3,3-tetraethoxypropane. Reagents were obtained from Sigma (St. Louis, MO, USA). TBARS levels were expressed as mg MA/kg sample.

### Volatile basic nitrogen evaluation

Volatile basic nitrogen (VBN) levels were assessed via the micro-diffusion method [18]. Each sample (5 g) was homogenized with 45 mL of distilled water for 2 min at 8,000 rpm and subsequently filtered through Whatman No. 1 (Whatman International, Maidstone, UK) filter paper. One mL of the filtered sample solution and 1 mL of K_2_CO_3_ solution were placed on the outer section of a Conway micro-diffusion cell. Next, 1 mL of 0.01 N H_3_BO_3_ and 50 μL of indicator (0.066% methyl red in ethanol: 0.066% bromocresol green in ethanol = 1:1) were placed in the inner section. Cells were incubated for 90 min at 37°C and titrated with 0.02 N H_2_SO_4_ solution until a faint reddish color was visible.

### Residual nitrite content measurement

Cooked cured pork loin (10 g) was homogenized with 150 mL of heated distilled water for 2 min. The resulting mixture was added with 10 mL of 0.5 N sodium hydroxide and 10 mL 12% ammonium thiosulfate. After heating at 80°C for 20 min, the solution was added with 20 mL of ammonium acetate buffer and distilled water to a volume of 200 mL. After incubation for 10 min at room temperature (25°C), 1 mL of sulphanilamide solution, 1 mL of N-(1-naphythyl) ethylenediamine dihydrochloride reagent, and 3 mL of distilled water were added to 20 mL of the filtered solution. The absorbance at 540 nm was measured using a UV/Vis spectrophotometer (Optizen 2120 UV plus, Mecasys Co. Ltd., Daejeon, Korea). Standard curve was used for calculating residual nitrite content [19].

### Sensory evaluation

A ten-member panel consisting of trained researchers from the Department of Food Sciences and Biotechnology of Animal Resources at Konkuk University in Korea evaluated the properties of the cured pork loin samples from the different treatments. Each treatment was evaluated based on interior color (bright reddish color), flavor (typical cured meat flavor), off-flavor (unpleasant typical vegetable flavor), and overall acceptability. Cured pork loin samples were cooked at 75°C for 30 min, cooled at 25°C for 60 min and cut into 1×1×1 cm. Six samples were served randomly to the panelists in each sessions with random three-digit numbers (6 samples were served in one session) and session was proceed for three times to evaluate sensory. Sensory evaluations were performed under fluorescent lighting. Panelists were instructed to cleanse their palates between samples using water. The interior color (1 = extremely undesirable, 9 = extremely desirable), flavor (1 = extremely undesirable, 9 = extremely desirable), off-flavor (1 = extremely undesirable, 9 = extremely desirable), and overall acceptability (1 = extremely undesirable, 9 = extremely desirable) of the cooked samples were evaluated using a nine-point descriptive scale. This analysis was conducted using the Hedonic test described by Bergara-Almeida et al [20].

### Statistical analysis

The entire test was done in triplicate and there was no significant difference between replicates (p>0.05). Results are presented as mean values and standard deviation in tables. All statistical analyses were analyzed as completely randomized design using SPSS Ver. 20.0 (SPSS Inc., Chicago, IL, USA). One-way analysis of variance and Duncan’s multiple range tests were used to determine the significant differences among treatments (p<0.05) in chemical, physical, and sensory analysis. Nitrite, spinach juice and fermented spinach juice were considered as main effects. In sensory evaluation, split plot design was used for analyzing data. Treatments added with different nitrite type (sodium nitrite and natural nitrite) were considered as fixed effects and panelists and session were considered as random effects.

## RESULTS AND DISCUSSION

### Cooking loss and pH

The control and treatment pork loins showed no significant differences in cooking loss (p>0.05) (Table 2). Higher cooking loss has been demonstrated to be caused by thermal denaturation of proteins in the meat and meat products [21]. According to Krause et al [10], sliced cooked ham cured with natural nitrite showed no significant differences in cooking loss compared to ham cured with sodium nitrite. Similar results for the cured pork loins were observed in this study. Generally, value of cooking loss was increased when pH of meat was decreased and approached an isoelectric point of meat [22]. However, there were no difference between treatments in cooking loss. It might be derived from small difference in pH though it was statistically significant difference between treatments.

Samples cured with fermented spinach juice had lower pH values than those cured with spinach juice (p<0.05) in raw. These results were consistent with those of Kim et al [15], who reported that pork loin cured with fermented spinach had lower pH values than those of the control samples. During the fermentation step, spinach juice undergoes acidification because the starter culture produces lactic acid as metabolite [10]. Samples in the control, T1, and T3 treatments had lower pH values than those of samples in the T2 and T4 treatments (p<0.05), which is attributed to the high pH of brine-containing materials, such as spinach juice (pH 5.73). The pH values of the chemicals present in the formulations significantly affect the pH of the final product. Given that the pH of fermented spinach is similar to that of raw pork loin (pH 5.43), the addition of fermented spinach juice did not significantly affect the pH of raw cured pork loin (p<0.05). The pH of cooked cured pork loins was higher than those of raw cured pork loin because thermal protein denaturation increases the pH of meat products [15]. A similar trend in pH was observed between cooked and raw samples. However, statistical difference was not shown (p>0.05).

### Color

Table 3 shows the interior surface colors of the experimental samples. Myoglobin forms NO-myoglobin, which has a bright reddish color when combined with nitrite [7]. Before cooking, control (−) samples without nitrite showed the lowest redness values (p<0.05), and T3 samples, which represent cured pork loins with fermented spinach juice, had the highest redness values (p<0.05). For cooked condition, cured pork loins formulated with fermented spinach juice, which contained natural antioxidants, showed more intense red color values than those of the control (+) samples (p<0.05), since nitrosohemochrome can form easily when antioxidants are added to meat products [7,15]. A previous study showed that the compound use of synthetic and natural nitrites from fermented Swiss chard produced meat with higher redness values than meat cured with natural nitrite alone [23]. For cooked condition, our results showed that the combined use of synthetic nitrite and natural nitrite from spinach produced more pronounced effects on redness coupling of cured pork loin than that of synthetic nitrite alone (p<0.05). Direct addition of nitrite increased the curing efficiency because longer incubation periods are required by the microorganisms to reduce nitrate in spinach into nitrite and to react with myoglobin [11]. Cured pork loin containing pre-converted nitrite from spinach showed higher efficiency in reddish coupling than other treatments during the curing step (p<0.05). Hue angle serves a useful index for evaluating the intensity of the red color of meat. Larger values of the hue angle indicate less redness, less coupling reaction, and higher metmyoglobin [24,25]. Although raw cured pork loin added with nitrite had high hue angle values, cooked T1 and T3 samples had lower hue angles than those of control (+) samples (p<0.05). These results indicated that pre-converted nitrite from spinach has a more pronounced effect on the coupling of cured pork loin compared to synthetic nitrite.

### Thiobarbituric acid reactive substance and volatile basic nitrogen

**Figure 1:**
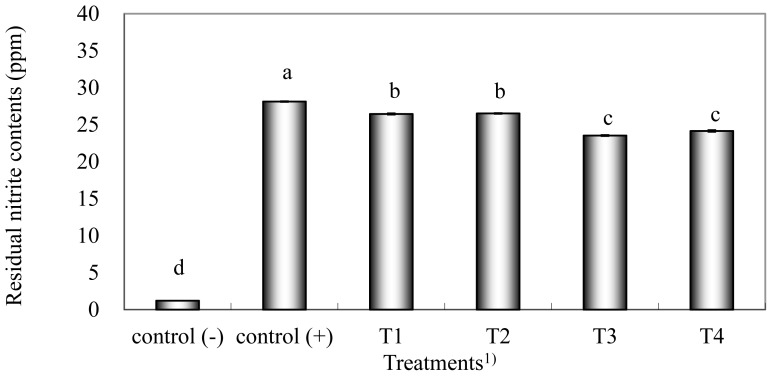
Effects of partial addition sodium nitrite on residual nitrite level of raw cured meat with spinach juice. ^a–d^ Means within a column with different letters are significantly different (p<0.05). 1) Control (−), curing meat with nitrite free brine; Control (+), curing meat with 0.08% nitrite in brine; T1, curing meat with fermented spinach juice in half brine; T2, curing meat with spinach juice in half brine with starter culture; T3, curing meat with fermented spinach juice; T4, curing meat with spinach juice with starter culture.

The TBARS and VBN values of cured pork loin samples are shown in Table 4. Control (−) samples had the highest TBARS values because nitrite acts as a very strong antioxidant when added to meat products [7]. Control (+), T1, and T3 samples directly added with nitrite showed no significant differences in TBARS values (p>0.05). In a previous study, meat products with vegetable powder contained low nitrite levels and showed reduced lipid oxidation, similar to samples added with high nitrite concentrations [26]. However, even though residual nitrite content (Figure 1) was not different between T1 and T2 and T3 and T4, T2 and T4 samples had higher TBARS values than those of T1 and T3 samples (p<0.05) because of the incubation step, metabolic activities of the microorganisms [10] and absence of nitrite that can inhibit lipid oxidation of meat during period of pickling. Therefore, nitrates from vegetable sources should be reduced to nitrite before the curing step. The above results were consistent with those of Sindelar et al [11], who compared meat products added with pre-converted nitrite and non-pre-converted nitrite. Incubation during the curing step was revealed to negatively affect lipid oxidation of meat products because of the protein decomposition, which could be caused by generation of heat or metabolic activity of the microorganisms [16]. The VBN value is an indicator of protein decomposition [27]. Kim et al [15] demonstrated that high VBN levels are caused by the metabolic activity of microorganisms in the fermented spinach juice or starter culture. In the present study, cured pork loin samples with pre-converted nitrite had lower VBN value than samples from the other treatments (p<0.05). Furthermore, samples from all treatments had higher VBN values than those in the control group (p<0.05).

### Residual nitrite content

Previous studies have suggested that residual nitrite in meat products are carcinogenic [9,13]. Antioxidants, such as ascorbate, can reduce residual nitrite in the meat products into nitric oxide [28]. Residual nitrite contents in samples are shown in Figure 1. Although added nitrite contents (sum of sodium nitrite and natural nitrite) was clearly same in Control (+), T1, and T3, Control (+) samples showed the highest residual nitrite contents (p<0.05). Treatments formulated with natural nitrite showed significantly lower residual nitrite contents (p<0.05) than control (+) and T3 had lower residual nitrite content than T1 (p<0.05), indicating that nitrite derived from natural sources can produce lower amounts of residual nitrite in meat products. Furthermore, in treatment that cured with spinach juice, T4 had lower residual nitrite contents than T2 (p<0.05). A previous study showed meat products added with natural nitrite from vegetable powder had lower residual nitrite contents than meat products added with synthetic nitrite alone [23,29]. Therefore, natural nitrite can be used to replace synthetic nitrite to produce meat products with similar quality characteristics and lower residual nitrite contents.

### Sensory evaluation

The effects of natural nitrite from spinach on sensory evaluation are shown in Table 5. All treatments except for the control (−) showed no significant differences in color evaluation scores (p<0.05). However, in terms of flavor, samples subjected to nitrite treatment showed lower scores than those in the control group (p<0.05) because of the typical flavor of vegetables. Many previous studies showed that natural nitrite from vegetables negatively affect the flavor of meat products [9,11,12,16,23]. Therefore, food manufacturers must develop methods to minimize the typical vegetable flavor found in meat products. Scores for off-flavor were also influenced by the presence of the vegetable flavor. However, T1 and T2, which were added with natural nitrite, showed no differences in off-flavor scores with those of control samples (p>0.05). Furthermore, T1 samples showed no difference in overall acceptability compared to those of controls (p>0.05). Tsoukalas et al [26] compared sensory parameters between samples containing natural and synthetic nitrites and reported similar results. Higher vegetable powder concentrations significantly reduced the overall acceptability of samples [30]. According to Shin et al [23], partial addition of natural nitrite can prevent the decrease in sensory evaluation of the meat products, which were consistent with the results of the present study. In conclusion, vegetable flavor negatively influences sensory evaluation of samples, which can be solved by the addition of natural nitrite to the formulation.

## CONCLUSION

Natural nitrites derived from spinach effectively improved the quality characteristics of cured pork loin. Combined synthetic and natural nitrites produced cured pork loin with better quality characteristics than those formulated with synthetic nitrite alone. However, given the typical flavors from vegetables, the sensory scores for cured pork loin samples with natural nitrite were lower than those of the control samples. Therefore, cured pork loins containing natural nitrites must be added with spices to remove the vegetable flavor from spinach.

## Figures and Tables

**Table 1:** Brine formulations for establishment of sodium nitrite substitution condition (unit: %)

Items	Control (−)	Control (+)	T1	T2	T3	T4
Water	92	91.92	45.98	45.86	-	-
Fermented spinach juice	-	-	45.98	-	92	
Spinach juice	-	-	-	45.86	-	91.75
Salt	8	8	8	8	8	8
Nitrite	-	0.08	0.04	0.04	-	-
Starter culture	-	-	-	0.25	-	0.25
Total	100	100	100	100	100	100

**Table 2:** Effects of partial addition of sodium nitrite on cooking loss and pH of cured meat with spinach juice

Items		Control (−)1)	Control (+)	T1	T2	T3	T4
Cooking loss (%)		31.03±0.62	32.84±0.66	33.13±1.58	34.66±0.95	33.15±2.77	33.36±1.02
pH	Raw	5.37±0.01^b^	5.37±0.01^b^	5.37±0.01^b^	5.45±0.01^a^	5.36±0.01^b^	5.45±0.01^a^
	Cooked	5.65±0.01	5.64±0.01	5.67±0.01	5.69±0.01	5.66±0.01	5.67±0.01

All values are mean±standard deviation of three replicates (n = 3).

1) Control (−), curing meat with nitrite free brine; Control (+), curing meat with 0.08% nitrite in brine; T1, curing meat with fermented spinach juice in half brine; T2, curing meat with spinach juice in half brine with starter culture; T3, curing meat with fermented spinach juice; T4, curing meat with spinach juice with starter culture.

a,b Means within a row with different letters are significantly different (p<0.05).

**Table 3:** Effects of partial addition of sodium nitrite on color of cured meat with spinach juice

Items	Control (−)1)	Control (+)	T1	T2	T3	T4
Raw						
L*	45.87±0.32^c^	44.14±0.96^d^	44.62±0.65^d^	46.89±0.67^b^	49.57±0.52^a^	44.73±0.34^b^
a*	7.71±0.24^c^	8.71±0.16^b^	8.89±0.29^b^	9.16±0.27^ab^	9.41±0.54^a^	9.02±0.61^ab^
b*	3.93±0.94^c^	5.32±0.19^b^	5.27±0.47^b^	4.65±0.13^b^	5.93±0.22^a^	4.77±0.25^b^
H°	26.90±0.83^b^	30.43±2.14^a^	30.58±1.50^a^	30.58±1.50^a^	26.91±0.65^b^	27.91±0.99^b^
ΔE	-2)	2.62±0.83	2.62±0.74	2.30±1.09	4.04±1.61	2.20±1.03
ΔC	-	1.58±0.49	1.85±0.64	1.67±0.55	2.49±0.61	1.61±0.66
Cooked						
CIE L*	73.52±0.94^c^	76.17±0.11^a^	68.74±0.55^e^	75.30±0.13^b^	70.09±1.01^d^	74.60±0.18^b^
CIE a*	1.72±0.39^d^	8.37±0.17^c^	11.46±0.05^a^	8.33±0.09^c^	10.99±0.58^b^	8.16±0.05^c^
CIE b*	9.00±0.39^a^	5.98±0.11^d^	6.86±0.27^b^	6.37±0.19^c^	6.52±0.12^c^	6.01±0.05^d^
H°	78.67±0.90^a^	35.53±0.56^b^	31.07±0.69^c^	37.41±0.56^b^	30.72±0.93^c^	36.37±0.15^b^
ΔE	-	7.75±0.33^c^	10.94±0.31^a^	7.32±0.25^c^	10.20±0.97^b^	7.16±0.16^c^
ΔC	-	7.24±0.17^b^	9.82±0.18^a^	7.05±0.06^b^	9.53±0.62^a^	7.04±0.06^b^

All values are mean±standard deviation of three replicates (n = 3).

1) Control (−), curing meat with nitrite free brine; Control (+), curing meat with 0.08% nitrite in brine; T1, curing meat with fermented spinach juice in half brine; T2, curing meat with spinach juice in half brine with starter culture; T3, curing meat with fermented spinach juice; T4, curing meat with spinach juice with starter culture.

2) Values of control ΔE and ΔC were used in the calculation of other treatments. Hence, no value was given to that column.

a–e Means within a row with different letters are significantly different (p<0.05).

**Table 4:** Effects of partial addition sodium nitrite on volatile basic nitrogen and thiobarbituric acid reactive substance of cured meat with spinach juice

Items	TBARS (mg MD/kg meat)	VBN (mg%)
Control (−)1)	0.88±0.02^a^	14.81±0.55^d^
Control (+)	0.08±0.01^d^	16.51±1.08^c^
T1	0.06±0.01^d^	18.77±0.56^b^
T2	0.10±0.01^c^	21.56±1.06^a^
T3	0.08±0.01^d^	19.04±0.65^b^
T4	0.30±0.01^b^	21.83±0.90^a^

All values are mean±standard deviation of three replicates (n = 3).

TBARS, thiobarbituric acid reactive substance; VBN, volatile basic nitrogen.

1) Control (−), curing meat with nitrite free brine; Control (+), curing meat with 0.08% nitrite in brine; T1, curing meat with spinach juice in half brine; T2, curing meat with spinach juice in half brine with starter culture; T3, curing meat with fermented spinach juice; T4, curing meat with spinach juice with starter culture.

a–d Means within a column with different letters are significantly different (p<0.05).

**Table 5:** Effects of partial addition sodium nitrite on sensory evaluation of raw cured meat with spinach juice

Items	Control (−)1)	Control (+)	T1	T2	T3	T4
Color2)	6.00±1.07^b^	7.63±0.74^a^	7.38±0.92^a^	7.50±0.53^a^	7.00±0.76^a^	7.13±0.83^a^
Flavor	6.75±0.46^ab^	7.13±0.35^a^	6.50±0.53^b^	6.00±1.31^bc^	6.13±0.64^bc^	5.38±0.92^c^
Off-flavor	6.50±0.76^a^	7.00±0.41^a^	6.50±0.53^ab^	6.25±1.04^ab^	6.13±0.64^b^	5.25±1.04^c^
Overall acceptability	6.50±0.93^ab^	7.13±0.35^a^	6.63±0.74^ab^	5.88±1.25^bc^	6.13±0.83^bc^	5.43±0.79^c^

All values are mean±standard deviation of three replicates (n = 3).

1) Control (−), curing meat with nitrite free brine; Control (+), curing meat with 0.08% nitrite in brine; T1, curing meat with fermented spinach juice in half brine; T2, curing meat with spinach juice in half brine with starter culture; T3, curing meat with fermented spinach juice; T4, curing meat with spinach juice with starter culture.

2) Color (1 = extremely undesirable, 9 = extremely desirable), Flavor (1 = extremely undesirable, 9 = extremely desirable, typical cured meat flavor), Off-flavor (1 = extremely undesirable, 9 = extremely desirable, typical vegetable flavor), and overall acceptability (1 = extremely undesirable, 9 = extremely desirable).

a–c Means within a row with different letters are significantly different (p<0.05).
